# NHC Backbone Configuration in Ruthenium-Catalyzed Olefin Metathesis

**DOI:** 10.3390/molecules21010117

**Published:** 2016-01-20

**Authors:** Veronica Paradiso, Chiara Costabile, Fabia Grisi

**Affiliations:** Dipartimento di Chimica e Biologia “Adolfo Zambelli”, Università di Salerno, Via Giovanni Paolo II 132, Fisciano I-84084, Salerno, Italy; vparadiso@unisa.it (V.P.); ccostabile@unisa.it (C.C.)

**Keywords:** metathesis, NHC ligands, stereogenic centers, ruthenium catalysts

## Abstract

The catalytic properties of olefin metathesis ruthenium complexes bearing *N*-heterocyclic carbene ligands with stereogenic centers on the backbone are described. Differences in catalytic behavior depending on the backbone configurations of symmetrical and unsymmetrical NHCs are discussed. In addition, an overview on asymmetric olefin metathesis promoted by chiral catalysts bearing *C*_2_-symmetric and *C*_1_-symmetric NHCs is provided.

## 1. Introduction

Ruthenium-catalyzed olefin metathesis represents nowadays an indispensable synthetic tool for constructing carbon-carbon double bonds in both organic and polymer chemistry [1,2,3,4,5,6,7,8]. The use of *N*-heterocyclic carbenes (NHCs) as ancillary ligands [9,10] for ruthenium olefin metathesis complexes (second generation catalysts) has led to tremendous advances in the design of robust and effective catalysts for various metathesis applications, including some challenging and difficult transformations [11,12,13,14,15].

**Figure 1:**
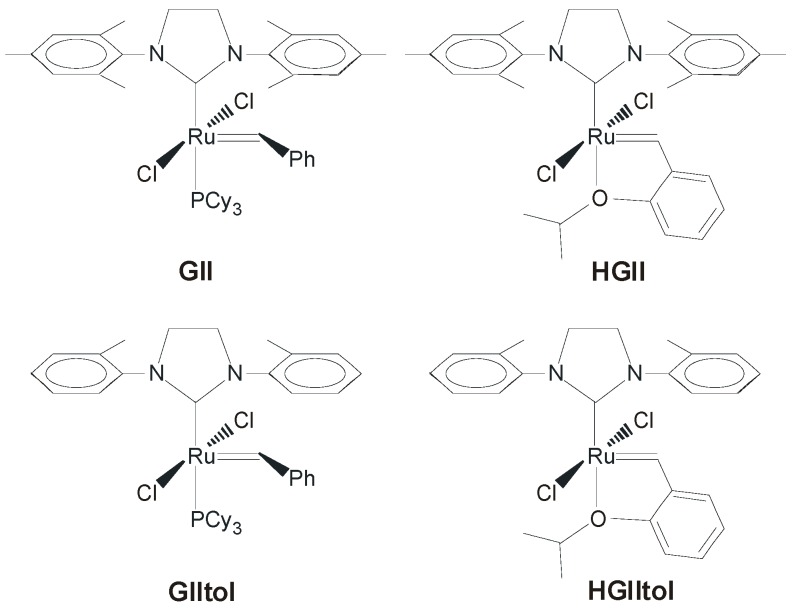
Commercial Grubbs’ and Hoveyda-Grubbs’ second generation catalysts.

In order to improve catalytic performance of NHC-stabilized ruthenium metathesis complexes, many efforts have been devoted to the manipulation of the NHC scaffold of the commercially available second generation catalysts (Figure 1).

Modifications of the NHC ligand include the nature of the ring backbone (saturated or unsaturated), substitution at the nitrogen atoms and at the carbon atoms of the backbone, ring-size variation, introduction of heteroatoms in the skeleton, and introduction of chirality [11,12,13,14,15]. Among these, modifications of the steric and electronic properties of substituents on the backbone and/or the nitrogen atoms have had a significant impact on catalyst activity, stability and selectivity in several metathesis applications [16,17,18,19,20,21,22,23,24,25,26,27,28,29].

This review provides an overview of the reactivity and selectivity shown by olefin metathesis ruthenium catalysts bearing NHC ligands with substituents on the backbone in definite stereochemical arrangements, mostly focusing on their catalytic performances in challenging metathesis reactions, such as asymmetric or sterically hindered reactions. Relevant literature data for ruthenium catalysts with *syn* and *anti* NHC backbone configurations are discussed according to the substitution pattern at the nitrogen atoms (symmetrical or unsymmetrical), highlighting, where it is possible, the effect of changing NHC backbone configuration on catalytic behavior. A brief description of ruthenium catalysts coordinated with backbone-monosubstituted NHCs is also presented.

## 2. Ruthenium Catalysts Bearing Symmetrically *N*-Substituted NHCs with *syn* or *anti* Backbone Configurations

### 2.1. Non-Aromatic N-Substituents

**Figure 2:**
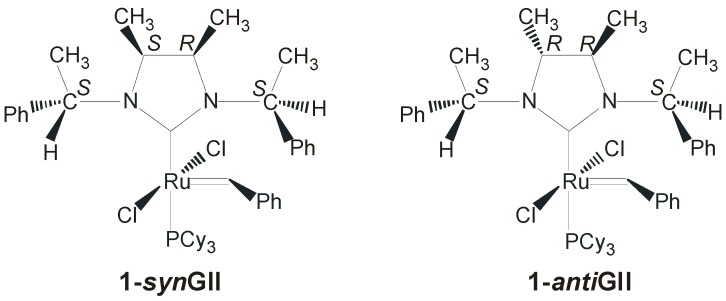
NHC-Ru complexes with *N*-(*S*)-phenylethyl groups and *syn* or *anti* methyl groups on the backbone.

Many research efforts have been devoted to improving catalyst performance by increasing the σ-donor properties of the NHC ligand through the introduction of more electron-donating *N*-alkyl substituents. However, most ruthenium complexes bearing only alkyl side chains were unstable and did not provide better catalytic results compared with the parent catalysts **GII** and **HGII** [30,31,32,33]. In 2008, the first examples of monophosphine Ru complexes bearing monodentate saturated NHC ligands which combine benzyl chiral groups on the nitrogens with methyl backbone substituents were reported in the literature (**1-*syn*GII** and **1-*anti*GII**, Figure 2) [21].

**Scheme 1:**
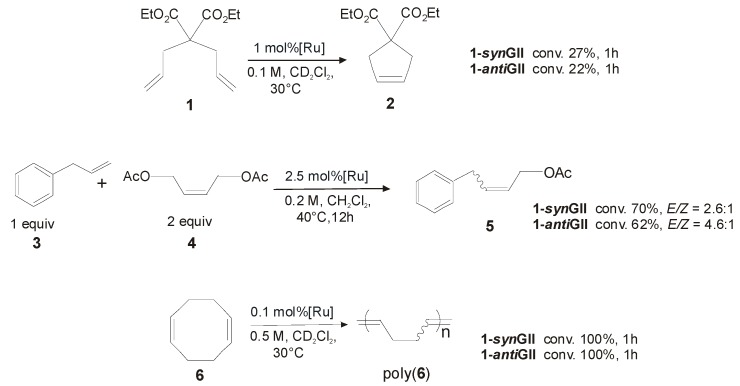
Representative RCM, CM and ROMP metathesis reactions.

Both complexes, presenting *S*-phenylethyl *N*-substituents and *syn* (**1-*syn*GII**) or *R*,*R-anti* (**1-*anti*GII**) methyl groups on the backbone, were prepared in around 40% yield by deprotonation of the corresponding imidazolinium salts obtained after condensation of the corresponding chiral diamines. **1-*syn*GII** showed higher cataytic activity than **1-*anti*GII** in all reported representative reactions (Scheme 1) [34], however both performed worse than **GII**.

Indeed, in the presence of **1-*syn*GII** the ring-closing metathesis (RCM) of diethyl diallylmalonate (**1**) led to only 27% of conversion to product **2** after 1 h, and the cross–metathesis (CM) of allylbenzene (**3**) and *cis*-1,4-diacetoxy-2-butene (**4**) to 70% yield of **5** in 12 h, giving in all reactions slightly higher conversion than its *anti* analogue. Better results, although mediocre compared to **GII**, were observed in the ring-opening metathesis polymerization (ROMP) of **6**, where 100% conversion was achieved in 1 h. Most likely the presence of benzyl *N*-substituents slows down the efficiency of catalysts with respect to *N*-aryl groups. Nevertheless, it is possible to discriminate in the described complexes a different behavior of catalysts with *syn* or *anti* NHC backbone configuration, being **1-*syn*GII** always better performing than **1-*anti*GII**. Interestingly, in ROMP and CM reactions both the catalysts gave improved selectivity toward *Z* double-bond formation (*E*/*Z* ratio of 2.6:1 with **1-*syn*GII**).

### 2.2. N-Aryl Substituents

**Figure 3:**
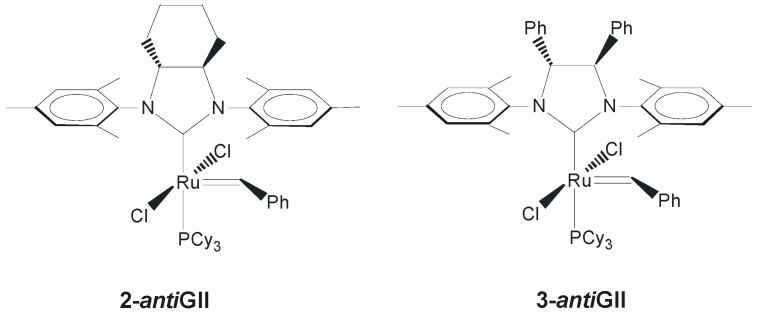
Catalysts with *anti* NHC backbone.

The first complexes with modifications of the backbone of the NHC ring (**2-*anti*GII** and **3-*anti*GII**, Figure 3) were reported by Grubbs and coworkers in 1999, along with the famous second-generation catalyst **GII** [35]. The introduction of this type of NHCs was essentially due to an expected enhanced activity of the resulting ruthenium catalysts, as a consequence of the more basic nature of the NHC, presenting a saturated backbone, with respect to the ruthenium-based complexes with unsaturated NHCs known until then. The RCM activity of complexes **2-*anti*GII** and **GII** was explored and an increased reactivity especially at elevated temperatures was actually found.

**Figure 4:**
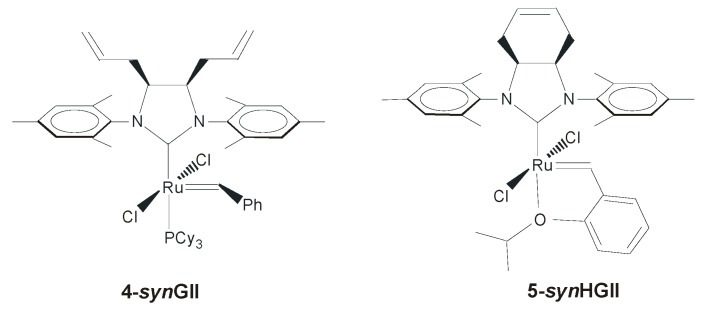
Complexes with olefinic moieties on the NHC.

The first example of a ruthenium complex bearing an NHC ligand with a *syn* configuration of the backbone was reported by Köhler *et al.*, in 2005 (Figure 4) [36]. With the aim of synthesizing complex **4-*syn*GII** with *syn* allyl substituents on the backbone as functional groups for immobilization of the catalyst to a solid support, they instead formed a new NHC ligand featuring an olefinic group in the ligand backbone, then employed it to prepare the complex **5-*syn*HGII**. In the ring-closing metathesis of *N,N*-diallyl-4-methylbenzenesulfonamide, complex **5-*syn*HGII** was slightly less active than the benchmark catalysts **HGIItol**, especially at low catalyst loadings.

**Figure 5:**
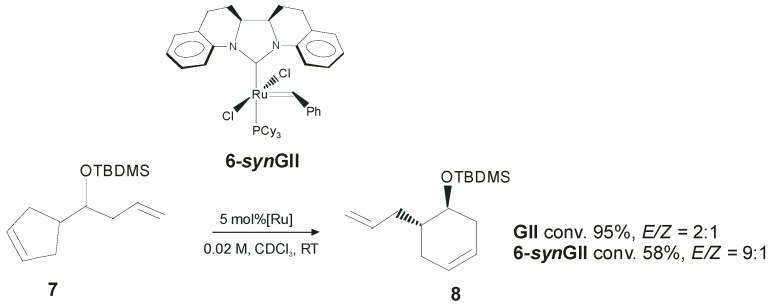
Diastereoselective RRM of **7** with catalyst **6-*syn*GII.**

In 2007 Blechert and coworkers presented a new ruthenium complex **6-*syn*GII** (Figure 5) containing an NHC ligand in which the *N*-aryl substituents were connected to the NHC backbone through a *syn* related C2 unit [37]. In this complex, the steric influence exerted by the aromatic moiety on the ruthenium alkylidene moiety is much stronger than in **GII**, therefore an increase of the diastereoselectivity of ring rearrangement metathesis reactions (dRRM) was expected. Catalyst **6-*syn*GII** was found to have limited stability in solution, even in the absence of olefin substrates, thus showing less catalytic efficiency than **GII**. Nevertheless, promising results in the RRM of racemic **7** were observed. Possible deactivation pathways involving intramolecular carbene-arene bond formation (intramolecular C-H insertion) were also investigated.

**Figure 6:**
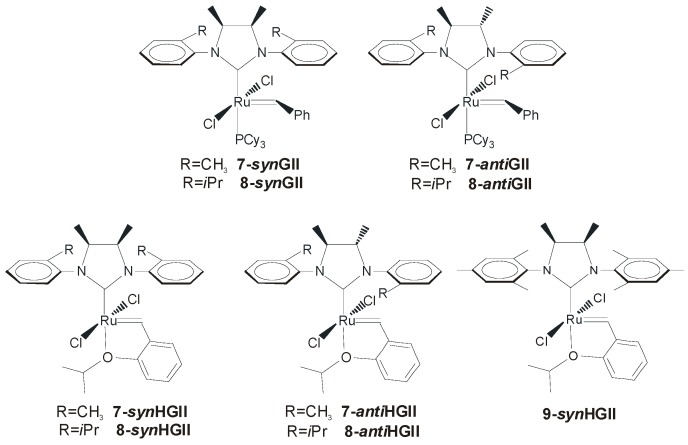
Catalysts with *syn* and *anti* methyl groups on the NHC backbone.

In 2009, the effect of NHC backbone configurations in ruthenium catalysts bearing aromatic *N*-tolyl groups was explored by Grisi and coworkers (**7-*syn*GII** and **7-*anti*GII**, Figure 6) [26]. The synthesis of **7-*syn*GII** and **7-*anti*GII** proceeded in good yield (55%–60%), although it required several steps, including the preparation of *meso* and chiral 1,2-diamines [38] to achieve the corresponding NHC ligand precursors.

The catalytic properties of **7-*syn*GII** and **7-*anti*GII** were probed in RCM reactions and intriguing results were registered above all in the RCM of hindered olefins carried out with **7-*syn*GII**. In fact, while **7-*syn*GII** promoted the RCM of **1** with almost the same efficiency of the commercial **GIItol** and slower rate than **GII** (Table 1), catalytic activity higher than that of **GIItol** and quite similar to **GII** was observed in the RCM of **9** (Table 2), and, more significantly, **7-*****syn*****GII** outperformed both **GIItol** and **GII** in the RCM of challenging encumbered substrate **11** (Table 3). **7-*anti*GII** showed a lower activity than **7-*syn*GII** in the RCM of all three malonate derivatives, with an enhanced gap for hindered substrates where only half of yield with respect to **7-*syn*GII** was reached in the ring-closure of **11**.

**Table 1:** RCM of diethyl diallylmalonate **1**. 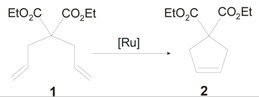

Catalyst	t (min)	Yield (%) ^a^	Ref.	Catalyst	t (min)	Yield (%) ^b^	Ref.
**GII**	40	>98	[34]	**HGIItol**	3	>99	[39]
**GIItol**	60	98	[26]	**7-synHGII**	4	>99	[26]
**7-synGII**	60	98	[26]	**7-antiHGII**	5	>99	[27]
**7-antiGII**	60	92	[26]	**8-synHGII**	3	>99	[27]
**8-synGII**	13	100	[27]	**8-antiHGII**	6	>99	[27]
**8-antiGII**	60	>96	[27]				

^a^ Reactions conducted in CD_2_Cl_2_ (0.1 M) at 30 °C, catalyst 1 mol %; ^b^ Reactions conducted in C_6_D_6_ (0.1 M) at 60 °C, catalyst 1 mol %.

**Table 2:** RCM of diethyl allylmethallylmalonate **9**. 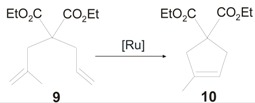

Catalyst	t (min)	Yield (%) ^a^	Ref.	Catalyst	t (min)	Yield (%) ^b^	Ref.
**GII**	60	98	[34]	**HGIItol**	8	>99	[39]
**GIItol**	60	84	[26]	**7-synHGII**	9	>99	[27]
**7-synGII**	60	90	[26]	**7-antiHGII**	16	>99	[27]
**7-antiGII**	60	80	[26]	**8-synHGII**	6	>99	[27]
**8-synGII**	20	95	[27]	**8-antiHGII**	12	>99	[27]
**8-antiGII**	60	83	[27]				

^a^ Reactions conducted in CD_2_Cl_2_ (0.1 M) at 30 °C, catalyst 1 mol %; ^b^ Reactions conducted in C_6_D_6_ (0.1 M) at 60 °C, catalyst 1 mol %.

**Table 3:** RCM of dietyl dimethallylmalonate **11**. 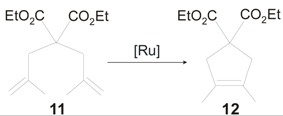

Catalyst	t (h)	Yield (%) ^a^	Ref.	Catalyst	t (h)	Yield (%) ^b^	Ref.
**GII**	96	17	[34]	**HGIItol**	0.5	>95	[39]
**GIItol**	1	70	[26]	**7-synHGII**	0.5	>95	[27]
**7-synGII**	1	82	[26]	**7-antiHGII**	1	78	[27]
**7-antiGII**	1	47	[26]	**8-synHGII**	1	85	[27]
**8-synGII**	1	70	[27]	**8-antiHGII**	1	59	[27]
**8-antiGII**	1	35	[27]	**9-synHGII**	72	55	[22]

^a^ Reactions conducted in CD_2_Cl_2_ (0.1 M) at 30 °C, catalyst 5 mol %; ^b^ Reactions conducted in C_6_D_6_ (0.1 M) at 60 °C, catalyst 5 mol %.

No significant difference between the two isomers of **7** with dissimilar backbones was appreciated in CM of **3** and **4**, being both catalysts less active than **GII**, with slightly higher *E* selectivity. The behavior of the two catalysts was also flattened in the ROMP of **6**, where both catalysts exhibit high activities as well as *E/Z* ratios very similar to **GII** and **GIItol** [26].

**Scheme 2:**
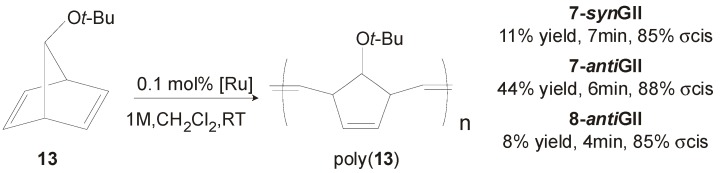
ROMP of **13** promoted by catalysts **7-GII**, and **8-*anti*GII**.

**7-*syn*GII** and **7-*anti*GII** were tested in the ROMP of **13** (Scheme 2), as well. Both catalysts gave highly sindiotactic poly(**13**) with low polydispersities (PDI = 1.1–1.2), 100% of *anti* units and *cis* contents that reached 88% in the case of **7-*anti*GII**. The latter surprisingly was found more active than the *syn* analogue, converting 44% of monomer in 6 minutes with 0.1% of catalyst [40].

An important role on the catalytic activity is also played by the bulkiness of *N*-aryl substituents, as generally reported for Ru complex, even with backbone unsubstituted NHCs [41,42,43,44,45]. Phosphine-containing Ru catalysts bearing *N-o*-isopropylphenyl groups in place of *N-o*-tolyl (**8-*syn*GII** and **8-*anti*GII,** Figure 6) can be prepared in good yields (60%–65%) with the same procedures adopted for **7-*syn*GII** and **7-*anti*GII** [27]. As expected by the increased catalyst bulkiness, **8-*syn*GII** and **8-*anti*GII** exhibited higher activity in the RCM of substrates **1** and **9** [41,42,43,44,45] with respect to the corresponding *o*-tolyl *N*-substituted catalysts (Table 1 and Table 2), whereas the RCM of the more encumbered **11** appeared slowed down (Table 3). The increase of catalyst bulkiness was counterproductive also in the ROMP of **13** (Scheme 2), where **8-*anti*GII** gave lower conversion to polymer (8%) with respect to its analogous with *N-o*-tolyl groups, **7-*anti*GII** (44%) [40].

The analogous phosphine-free catalysts **7-HGII** and **8-HGII** were easily synthesized with standard procedures in good yields (60%–88%) [27]. All obtained phosphine-free catalysts were able to convert **1** to **2** in a few minutes at 60 °C, with performances comparable to **HGIItol**, as emerged from data reported in Table 1. **7-*anti*HGII** and **8-*anti*HGII** revealed slightly less efficiency in the RCM of **9** (Table 2), and this gap was increased in the RCM of **11**, where only **7-*syn*HGII** showed an activity comparable with the commercial **HGIItol** (see data in Table 3).

The phosphine-free Ru catalyst bearing a *syn* methyl substituted backbone and *N*-mesityl groups (**9-*syn*HGII**, Figure 6), reported by Grubbs [22], is able to almost completely convert **1** in 1 h, at 30 °C in CD_2_Cl_2_ in the presence of 1 mol % of catalyst and 0.1 M of monomer, being less efficient than **HGII**. On the other hand at only 15 ppm catalyst loading and 50 °C, the catalyst performed slightly better than **HGII**, giving over 40% of conversion in 24 h. Less interesting behaviour was recorded for more encumbered substrates, and, in the RCM of **11**, even mediocre results were observed (55% conversion in 3 days, Table 3).

The role of the NHC backbone configuration in the RCM of challenging hindered substrates, investigated by theoretical studies on monophosphine *o*-tolyl *N*-substituted catalysts, revealed that the rate determining step occurs at the very beginning of the RCM, during the first CM of the substrate, and the corresponding transition state of the *syn* complex, that induces a *syn* orientation of the *o*-tolyl groups, presents lower energy than the *anti* complex mainly due to steric reasons [27].

The presence of NHC backbone substituents plays a significant additional role, besides the orientation of the *N*-aryl groups, that is the increase of catalyst stability toward decomposition through C-H activation by restricting rotation around the *N*-aryl bond [20,22].

**Scheme 3:**
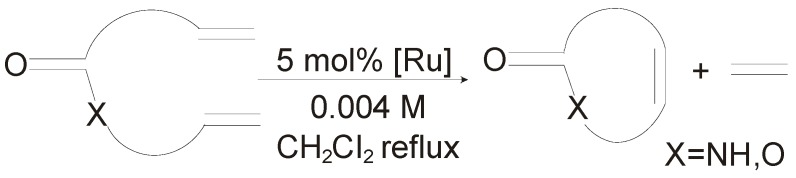
Macrocyclic RCM to form **14**–**17**.

Phosphine-containing catalyst **7-*syn*GII** was further tested in the macrocyclic RCM to produce unsaturated lactones and lactams according to the general reaction reported in Scheme 3.

**Figure 7:**
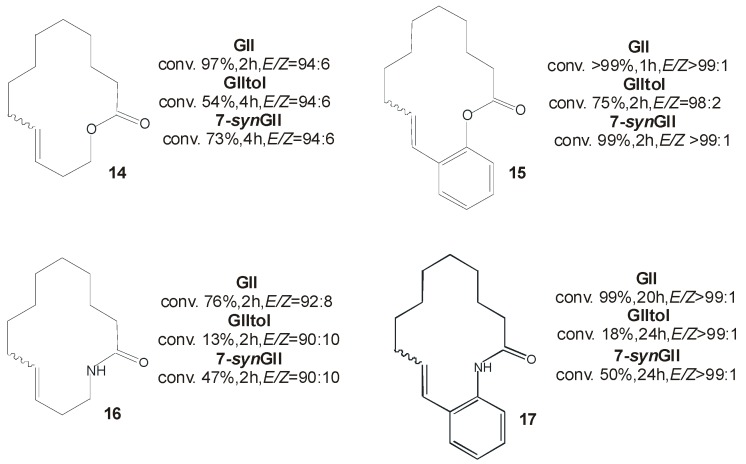
Macrocyclic RCM products **14**–**17**.

Results are collected in Figure 7, where comparison with the behavior of **GII** and **GIItol** is also reported. RCM of 14-membered lactones and lactams **14**–**17** were successfully carried out with **7-*syn*GII** that exhibited an intermediate activity in between **GII** and **GIItol** [46]. As for *E/Z* ratios of unsaturated macrocyclic products, they generally reflected the thermodynamic stabilities predicted by DFT calculations for the *E* and *Z* isomers. Only small differences, with slightly lower *E/Z* ratios observed, can be appreciated in the formation of **16** promoted by **7-*syn*GII** and **GIItol** (*E/Z* = 90:10).

**Figure 8:**
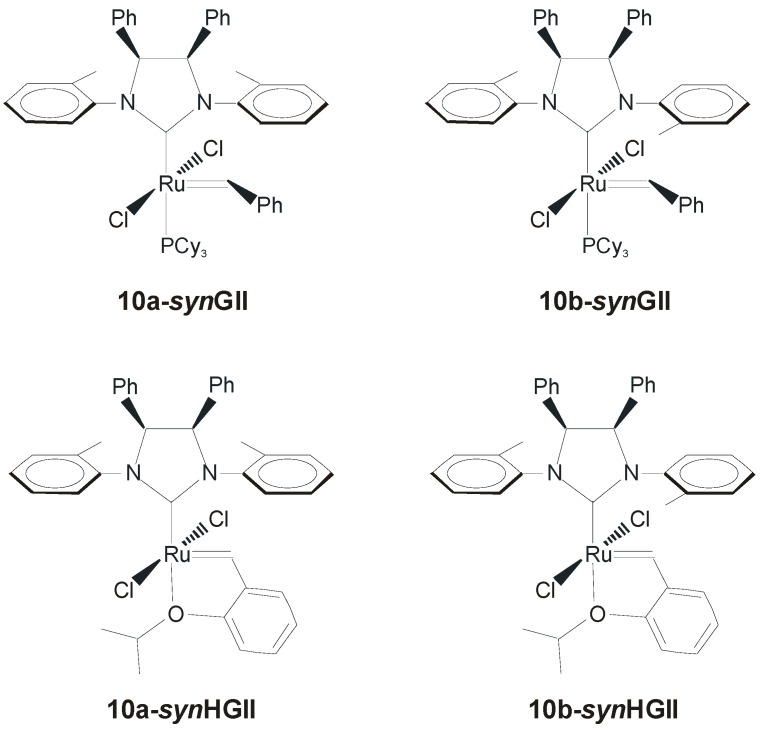
Catalysts with *syn* phenyl groups on the NHC backbone.

In another work, the same group presented ruthenium catalysts **10-*syn*GII** and **10-*syn*HGII** (Figure 8) where *syn* methyl substituents on the NHC backbone were replaced with more encumbered *syn* phenyl groups [47]. Complex **10-*syn*GII** was synthesized in good yields (58%) by a shorter synthetic pathway than that of **7-*syn*GII**, starting from the commercially available *meso*-1,2-diphenylethylenediamine. Surprisingly, two isomeric compounds, corresponding to different conformations of the *N*-tolyl substituents (**10a-*syn*GII** and **10b-*syn*GII,** Figure 8), were obtained.

Since complex **7-*syn*GII** as well as **GIItol** exist as a mixture of rotational isomers both in the solid state and in solution [18,26,48], the obtainment of separate, stable rotational isomers was strictly related to the steric pressure exerted by the bulky phenyl groups on the backbone. Phosphine-containing complexes **10a-*syn*GII** and **10b-*syn*GII** were easily converted in the corresponding phosphine-free catalysts **10a-*syn*HGII** (95% yield) and **10b-*syn*HGII** (84% yield) by treatment with 2-isopropoxystyrene.

The catalytic behavior of complexes with different *N*-tolyl conformations was investigated in model RCM reactions and, one year later, this study was extended to some other attractive RCM transformations, as well as other representative metathesis processes (ROMP, CM) [39]. Catalysts with frozen *syn* orientation of the *N*-tolyl groups were clearly identified as the most efficient in all the examined reactions, outperforming also the commercially available catalysts **GIItol** and **HGIItol** in almost all cases. In the challenging RCM of malonate derivative **11**, complex **10a-*syn*GII** gave the better result obtained with a phosphine-containing catalysts up to now (92% conversion within 30 min) (Table 4), proving to be more efficient than complexes **7-*syn*GII** and **GIItol** (Table 3). In comparison to the catalytic beaviour of complexes **7-*syn*GII** and **GIItol**, existing as a mixture of inseparable *syn* and *anti* NHC conformational isomers [18,26,48], the catalytic behavior of **10a-*syn*GII**, possessing frozen *syn* *N*-tolyl groups, furnished the unequivocal evidence for the importance of correctly disposed *N*-aryl groups to successfully accomplish RCM reactions. As for phosphine-free complex **10a-*syn*HGII**, although the differences in activity with respect to complexes **10b-*syn*HGII** and **HGIItol** were less pronounced (see Table 3 and Table 4), it turned out to be the most efficient, allowing the ring-closure of the difficult substrate **11** also at a low catalyst loading (0.5 mol %). In the easier RCM of hindered *N*-tosyl derivatives, such as *N*-allyl-4-methyl-*N*-(2-methylallyl)benzenesulfonamide and 4-methyl-*N*,*N*-bis(2-methylallyl)benzenesulfonamide, catalyst loadings as low as 0.05–0.1 mol % were required to achieve quantitative conversions [47,39].

**Table 4:** RCM of dienes **1**, **9** and **11** with catalysts **10a** and **10b**.

Diene Substrate	RCM Product	Catalyst ^a^ (mol %)	t (min)	Yield (%)	Catalyst ^b^ (mol %)	t (min)	Yield (%) ^b^
**1**	**2**	**10a-*syn*GII (1.0)**	30	>98	**10a-*syn*HGII (1.0)**	5	>99
**1**	**2**	**10b-*syn*GII (1.0)**	60	70	**10b-*syn*HGII (1.0)**	12	>99
**9**	**10**	**10a-*syn*GII (1.0)**	35	>95	**10a-*syn*HGII (1.0)**	6	>99
**9**	**10**	**10b-*syn*GII (1.0)**	60	66	**10b-*syn*HGII (1.0)**	60	95
**11**	**12**	**10a-*syn*GII (5.0)**	30	92	**10a-*syn*HGII (5.0)**	30	>99
**11**	**12**	**10b-*syn*GII (5.0)**	60	44	**10b-*syn*HGII (5.0)**	120	94

^a^ ref. [47]: reactions conducted in CD_2_Cl_2_ (0.1 M) at 30 °C; ^b^ ref. [47]: reactions conducted in C_6_D_6_ (0.1 M) at 60 °C.

In the RCM of (±)-linalool, a plant-derived monoterpene alcohol, to form 1-methylcyclopent-2-en-1-ol and isobutylene, the reactivity trend highlighted once again the superior performance of complex **10a-*syn*GII** with respect to both its *anti* conformer **10b** and **GIItol**. As an intriguing secondary aspect, both isomers of **10-*syn*GII**, as well as **GIItol**, were found able to promote the dehydration reaction of the cyclization product (1-methylcyclopent-2-en-1-ol) to well-defined mixtures of methylcyclopentadiene isomers, which represents a viable route to specialized fuel products.

The catalytic potential of **10-*syn*GII** and **10-*syn*HGII** was also explored in the ring-closing ene-yne metathesis (RCEYM) of (1-(allyloxy)prop-2-yne-1,1-diyl)dibenzene, where the overall reactivity profile puts complexes with *syn* related *N*-tolyl groups **10a-*syn*GII** and **10a-*syn*HGII** among the most efficient catalysts known until now.

**Figure 9:**
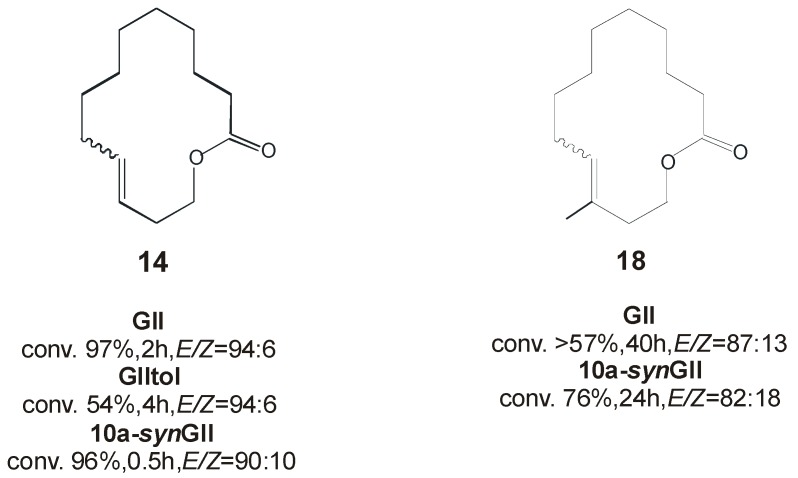
Macrocyclic RCM products **14** and **18**.

In the macrocyclic RCM reactions (Scheme 3) to form the 14-membered lactones **14** and **18**, respectively (Figure 9), complex **10a-*syn*GII** showed better performance than benchmark catalysts **GII** and **GIItol**, proving that correct *N*-tolyl conformation, combined with increased stability due to NHC backbone substitution, led to highly efficient catalysts.

The beneficial role of *syn* phenyl groups on the NHC backbone was also noticed in the CM of allyl benzene (**3**) and *cis*-1,4-diacetoxy-2-butene (**4**) and in the ROMP of 1,5-cyclooctadiene (**6**) (Scheme 1), where higher efficiency compared to commercial catalysts **GIItol** and **HGIItol** was found. No influence of the NHC backbone configuration on *E*/*Z* selectivity was instead observed in both the transformations.

Therefore, the judicious choice of *syn* related NHC backbone substituents permits the obtainment of stable Ru metathesis complexes with frozen *syn* NHC conformation, which seems to be a general requirement to successfully accomplish olefin metathesis reactions. This insight provides the inspiration for further development of NHC-bearing olefin metathesis catalysts.

## 3. Ruthenium Catalysts Bearing Unsymmetrically *N*-Substituted NHCs with *syn* and *anti* Backbone Configurations

**Figure 10:**
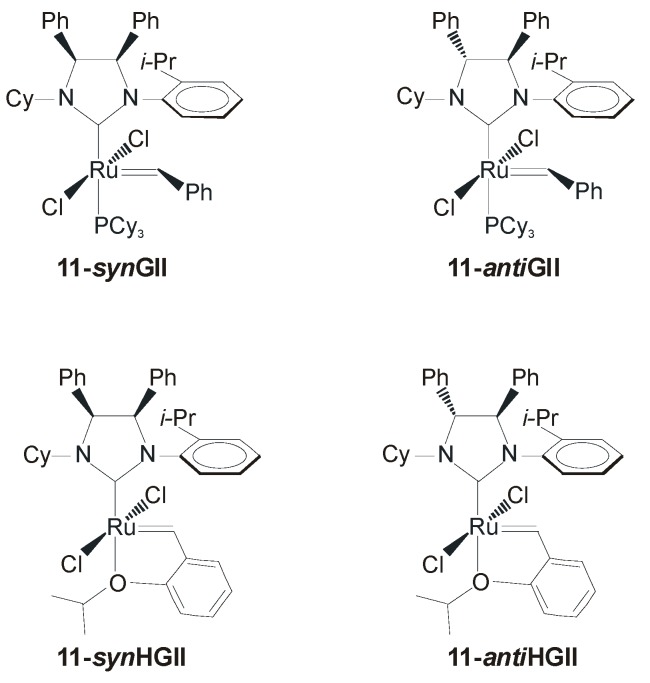
Catalysts with unsymmetrical NHCs and *syn* or *anti* phenyl substituted backbone.

The introduction of differently oriented substituents on the backbone of unsymmetrical NHCs was recently described by Grisi *et al.* [49]. The synthesis of ruthenium catalysts containing unsymmetrical NHCs that combine phenyl substituents on the backbone in *syn* or *anti* stereochemical relationships and *N*-cyclohexyl, *N*-isopropylphenyl groups (**11-GII** and **11-HGII**, Figure 10) was easily accomplished in moderate to good yields (45%–64%).

**Figure 11:**
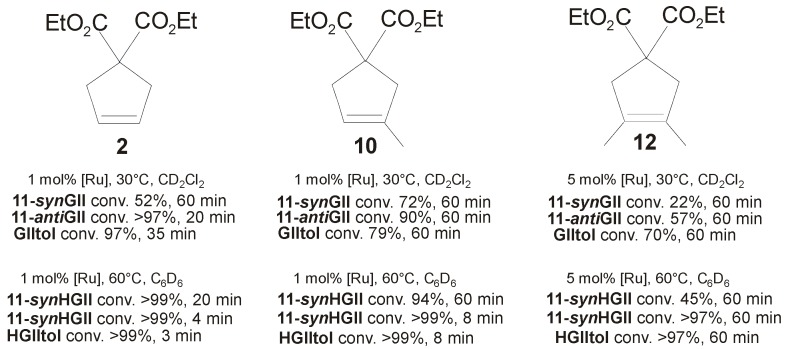
RCM to form cycloolefins **2**, **10** and **12**.

The catalytic properties of these complexes were evaluated in the RCM of diethyl diallylmalonate (**1**), diethyl allylmethallylmalonate (**9**), and diethyl dimethallylmalonate (**11**) to form cycloolefins **2**, **10** and **12** (Figure 11). Complexes **11-*anti*GII** and **11-*anti*HGII** performed better than their *syn* analogues in all of the tested RCM reactions. Notably, *anti* complexes disclosed an unexpectedly high propensity to the ring closure of the most hindered diolefins **9** and **11**, rivaling with the benchmark catalysts **GIItol** and **HGIItol**. This is in contrast with results reported for analogous systems incorporating symmetrical *N*-substituents [26,27], where instead *syn* isomers were found better performing, and suggests that the impact of backbone configuration of unsymmetrical *N-*substituted NHCs on catalyst properties is not easily predictable or interpretable.

The catalytic behavior of catalysts **11-GII** and **11-HGII** was also investigated in the CM of allylbenzene (**3**) and *cis*-1,4-diacetoxy-2-butene (**4**), depicted in Scheme 1. In this reaction, *syn* complexes **11-*syn*GII** and **11-*syn*HGII** showed better performances than their *anti* congeners, reaching high conversions (88% and 72%, respectively) and low E/Z ratios (~3).

Although preliminary, this study clearly indicates that the presence of differently oriented phenyl groups on the backbone of unsymmetrical NHCs can dramatically affect catalytic activity and selectivity, providing a new opportunity in catalyst design.

## 4. Ruthenium Complexes Bearing NHCs with *anti* Backbone Configuration in Asymmetric Metathesis

### 4.1. Symmetrical N-Substituents

**Figure 12:**
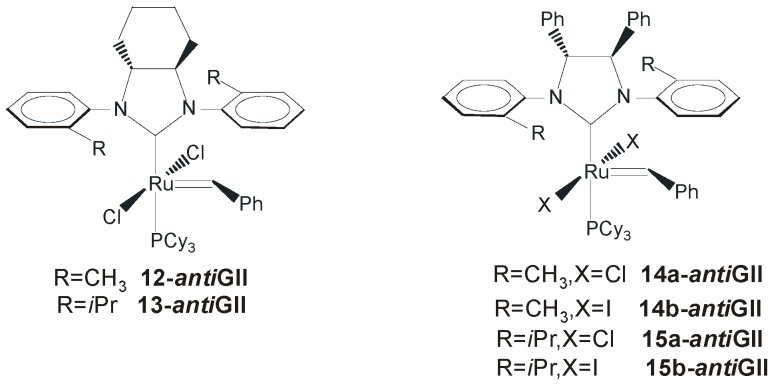
Catalysts **12-GII**–**15-GII** bearing monodentate NHCs with *anti* backbone.

The introduction of chirality into the backbone of the NHC framework was proposed for the first time by Grubbs and coworkers with the synthesis of ruthenium complexes **2-*anti*GII** and **3-*anti*GII** (Figure 3), bearing (1*R*,2*R*)-1,2-diaminocyclohexane and (1*R*,2*R*)-1,2-diphenylethylenediamine moieties as chiral entities [35]. Subsequently, in 2002, Grubbs’ group reported on the first asymmetric reaction promoted by these catalysts and by chiral catalysts **12-**, **13-**, **14-**and **15-*anti*GII** (Figure 12) [16], obtained in high yields (~70%) starting from commercially available enantiopure 1,2-diamines.

**Scheme 4:**
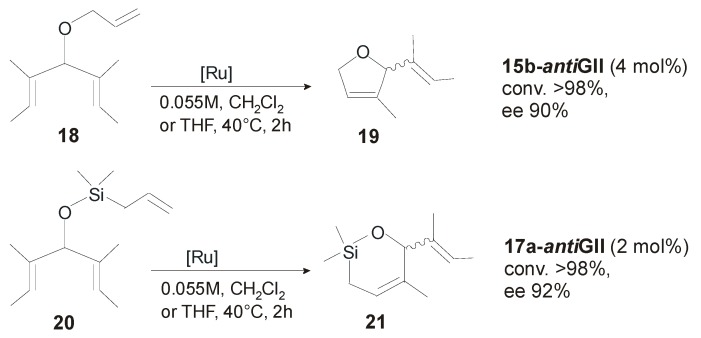
Asymmetric ring-closing metathesis reactions.

The catalytic behavior of all these complexes was evaluated in the asymmetric ring-closing metathesis (ARCM) of prochiral trienes (Scheme 4). Complexes derived from (1*R*,2*R*)-1,2-diphenylethylenediamine (**3-**, **14-** and **15-*anti*GII**) gave higher enantiomeric excesses than those prepared from (1*R*,2*R*)-1,2-diaminocyclohexane. Replacement of the mesityl substituent (in **2-** and **3-*anti*GII**) with *o*-tolyl (in **12-** and **14-*anti*GII**) or *o*-isopropylaryl groups (in **13-** and **15-*anti*GII**) led to increased enantioselectivity. More significantly, changing *in situ* the halide ligands of catalysts **12-GII**—**15-GII** from Cl^−^ to I^−^ further improved the enantioselectivity, allowing to reach 90% ee in the ARCM of triene **18** with catalyst **15b-*anti*GII** (Scheme 4). No effects of temperature and solvent on enantioselectivity were observed.

**Figure 13:**
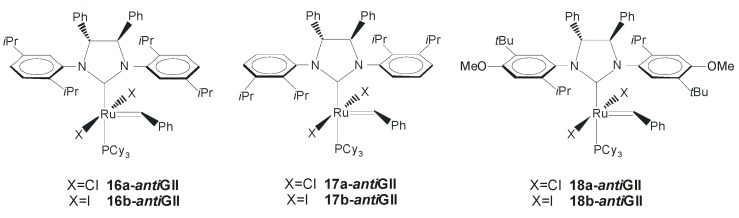
Catalysts **16-GII**–**18-GII** bearing monodentate NHCs with *anti* backbone.

The authors proposed that the chiral information of the NHC backbone was transferred to the ruthenium center through the *ortho*-substituted *N*-aryl groups (the so-called “gearing” effect), and, in a later study, a theoretical explanation of the origin of the enantioselectivity of the reaction was also offered by Costabile and Cavallo [50]. The authors rationalized the enantiomeric excesses experimentally obtained by Grubbs essentially as a result of the chiral folding of the *N*-bonded aromatic groups imposed by the *anti* Ph groups on the NHC backbone, which imposes a chiral orientation around the Ru=C bond, which, in turn, selects between the two enantiofaces of the substrate. To enhance enantioselectivity and expand the substrate scope of the ARCM, in 2006 Grubbs and coworkers reported chiral complexes **16-*anti*GII**–**18-*anti*GII**, differing for the number of substituents and/or for their arrangement on the *N*-aryl moieties (Figure 13) [51]. Among them, catalysts **16-GII** and **18-GII** presenting substitution on the *N*-aryl group of the NHC *para* to the *o*-isopropyl group showed enantioselectivities very similar to those of the parent chiral catalyst **15-GII**, while catalysts **17a-*anti*GII** and **17b-*anti*GII** with *o*-isopropyl substituents on the same side of the aromatic ring led to an increase in enantioselectivity.

**Scheme 5:**
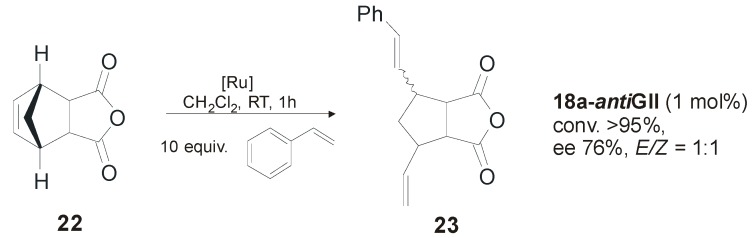
Asymmetric ring-opening cross metathesis.

Catalysts **15b-** and **17a-*anti*GII** were successfully employed in the desymmetrization of the alkenyl ether- and silyl ether-prochiral trienes forming five- to seven-membered rings. Conversions > 98% and 92% enantiomeric excess were obtained with **17a-*anti*GII** in the ARCM of silyl ether **20** (Scheme 4). In general, the diiodide catalysts showed higher enantioselectivity than the dichloride analogues, although in some cases lower conversions were obtained. The same type of catalysts was used by the Grubbs’ group in the asymmetric ring-opening cross-metathesis (AROCM) for a number of norbornenes and related strained bicycles [52]. In the model reaction of *cis*-5-norbornene-*endo*-2,3-dicarboxylic anhydride (**22**) with styrene (Scheme 5), catalyst **18a-*anti*GII** provided the product **23** with a good enantiomeric excess (76%) and high yield (95%) at low catalyst loading (1 mol %). No selectivity between *E* and *Z* isomers was observed, and the use of the analogous diiodide complex **18b-*anti*GII** generated *in situ* did not lead to significant improvements in reactivity or selectivity.

**Scheme 6:**
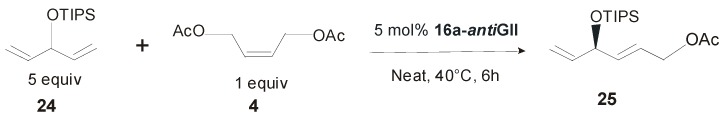
Asymmetric cross-metathesis.

Moreover, in the same work, the first examples for the most challenging asymmetric metathesis transformations, the asymmetric cross-metathesis (ACM) reactions, were reported. The enantiomeric excesses observed in ACM reactions of *meso*-diene substrates containing differently encumbered substituents at the allylic carbon atom with *cis*-1,4-diacetoxy-2-butene (**4**) were modest. In the ACM of TIPS-protected 1,4-pentadien-3-ol (**24**) with **4,** catalyst **16a-*anti*GII** gave **25** in 54% yield and 52% ee (Scheme 6).

Besides the above described catalysts with phenyl substituents on the backbone, also ruthenium complexes bearing symmetrical NHC ligands with *anti* methyl groups on the backbone were explored in the ARCM of model substrate **18** (Scheme 4). Catalyst **1-*anti*GII** [21], that bears a *C*_2_ symmetric NHC ligand with four stereogenic centers, since, in addition to the *R*,*R* methyl substituted backbone, they have *S*-phenylethyl *N*-substituents, gave modest enantioselectivity (33% ee) in the asymmetric ring-closure of **18**, probably due to the minor role of the chiral phenylethyl groups in transferring the asymmetric information from the backbone to the substrate.

In fact, when *o*-tolyl or *o*-isopropylaryl groups replaced phenylethyl groups on nitrogens, catalysts bearing NHCs with *S*,*S* methyl substituted backbone (**7-*anti*GII** and **8-*anti*GII**) [27] exhibited in the presence of NaI good enantioselectivities (83% ee and 90% ee, respectively), comparable to those obtained by analogous *anti* phenyl substituted backbone **14b-antiGII** and **15b-antiGII**.

### 4.2. Unsymmetrical N-Substituents

**Figure 14:**
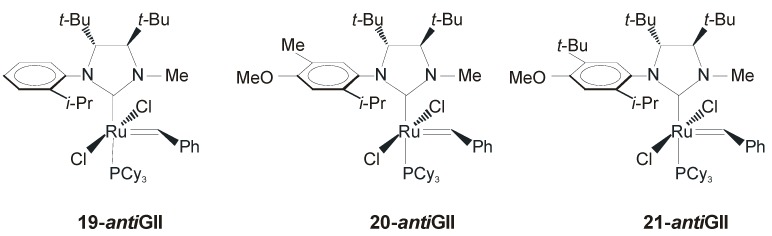
Catalysts bearing *C*_1_-symmetric monodentate NHC.

In 2007 Collins and coworkers reported the synthesis of the new complex **19-*anti*GII** bearing a C*_1_*-symmetric monodentate NHC ligand [53] (Figure 14). In this system the *anti* phenyl groups on the backbone were replaced with two *anti tert*-butyl groups, with the hope that an encumbered and electro-donating group could lead to an increased enentioselectivity and a pronounced reactivity. Considering the high steric hindrance, it seemed necessary to replace one *N*-aryl substituent with a smaller *N*-substituent, thus a Me-group was employed. Complex **19-*anti*GII** was obtained in low yield (~30%) starting from enantiopure 1,2-di-*tert*-butyldiamine.

For complexes bearing unsymmetrically substituted NHC ligands there are two possible rotational isomers: the *syn* isomer*,* in which the *N*-alkyl substituent lies over the carbene unit and its *anti* counterpart, in which the *N*-aryl substituent resides above the carbene. In the case of **19-*anti*GII**, NOE experiment and X-Ray analysis revealed the sole presence of the *syn* isomer.

Catalyst **19-*anti*GII** was tested in ARCM reactions of several prochiral trienes showing lower enantioselectivities with respect to Grubbs’ chiral catalysts **15a-** and **17a-*anti*GII** but, interestingly, the best results obtained in the RCM of standard substrate **18** were obtained without the use of any halide additive (Table 5) [54].

**Table 5:** RCM of prochiral triene **18**. 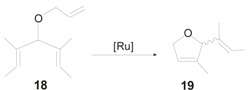

Catalyst	ee (%)	Conv. (%)	Ref.
**19-*anti*GII** ^a^	82	>98	[53]
**19-*anti*GII** ^b^	48	>98	[53]
**15a-*anti*GII** ^a^	35	>98	[51]
**15b-*anti*GII** ^b^	90	>98	[51]
**17a-*anti*GII** ^a^	46	>98	[51]
**17b-*anti*GII** ^b^	90	>98	[51]

^a^ Catalyst 2 mol %, CH_2_Cl_2_ (0.055 M), 40 °C; ^b^ Catalyst 4 mol % with NaI as the additive, THF, 40 °C.

**Figure 15:**
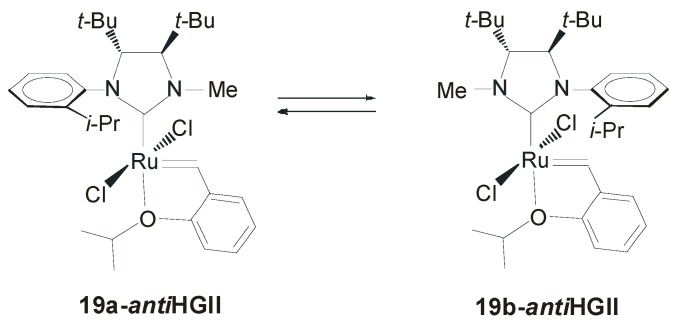
Rotational isomers of **19-*anti*HGII**.

However, the role of the halide addictive with catalysts **19-*anti*GII** is strongly dependent on the size of the ring formed. In fact, while in the ARCM of triene **18** a reduction of the enantioselectivity was observed by adding NaBr or NaI, when a seven-membered ring is involved the addition of the halide is even beneficial. The low enantiomeric excesses registered in desymmetization to form six- and seven-membered rings was attributed to the NHC rotation that could occur during the catalytic cycle causing the erosion of enantiomeric excesses. NHC rotation at room temperature was observed in **19-*anti*HGII**, the Hoveyda version of **19-*anti*GII**, that was formed as a mixture of rotational isomers with clear prevalence of the *syn* isomer (**19a-*anti*HGII**, Figure 15). Notwithstanding, **19-*anti*HGII** showed the same reactivity profile and enantioselectivities as **19-*anti*GII**, suggesting that, although the NHC is rotating at room temperature, the reaction takes place in the conformation in which the *N*-methyl group is *syn* to the carbene at a much faster rate than when the *N*-aryl group is *syn* to the carbene.

**Scheme 7:**
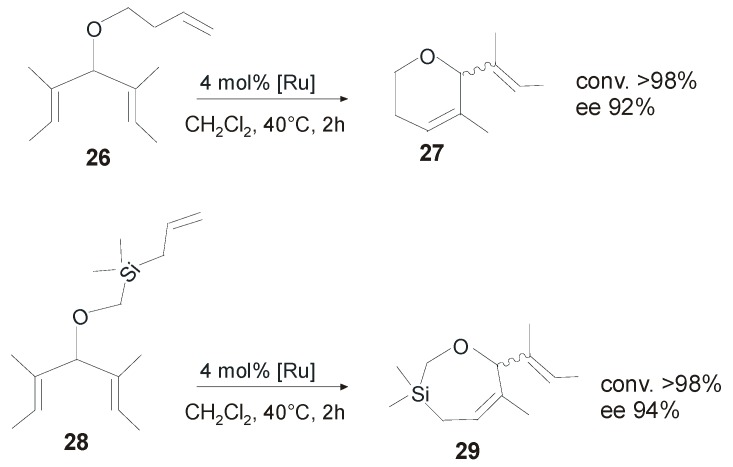
Asymmetric ring closing metathesis of **26** and **28** catalyzed by **21-*anti*GII**.

The same group also reported catalysts **20-*anti*GII** and **21-*anti*GII** (Figure 14), bearing a modified *N*-aryl substituent, with the expectation that an increased substitution at the *N*-aryl group would result in a hindered rotation and therefore in a reduction of the supposed interconversion between isomeric active species during the catalytic cycle [54]. **20-*anti*GII** and **21-*anti*GII** were obtained in moderate yields (42%–44%) as a mixture of rotational isomers, in which the *syn* isomer prevailed. No rotation of the NHC ligand was observed in either catalyst at room temperature. Catalysts **20-*anti*GII** and **21-*anti*GII** showed similar or enhanced catalytic performances in terms of both activity and selectivity with respect to the parent catalyst **19-*anti*GII**. Therefore, all the significant reactivity of **20-** and **21-*anti*GII** was assumed to occur from the major *syn* isomer. Notably, the high levels of asymmetric induction observed in ARCM reactions were achieved without the use of halide additives and, in particular, catalyst **21-*anti*GII** was found to be especially efficient in the asymmetric ring closing metathesis involving six and seven-membered rings (Scheme 7). All the catalysts were also able to promote the asymmetric synthesis of [7] helicene. Using vinylcyclohexane as an additive and C_6_F_6_ as a solvent, **21-*anti*GII** gave 80% of enantiomeric excess [55].

**Scheme 8:**
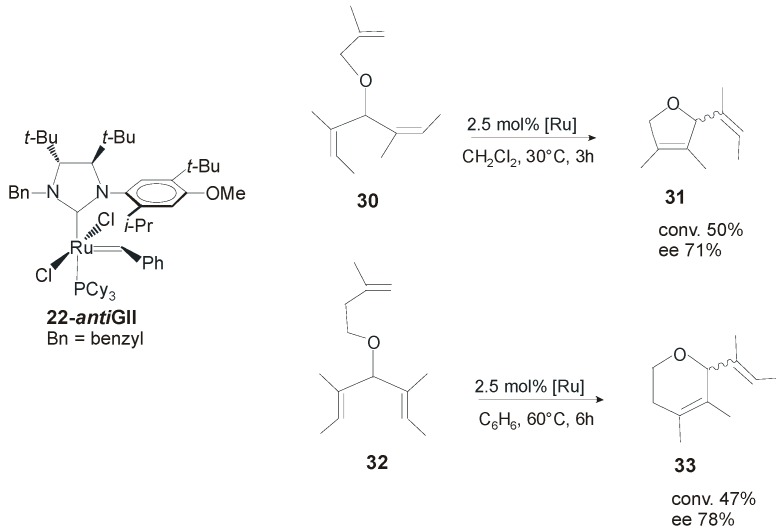
Asymmetric ring closing metathesis of **30** and **32** catalyzed by **22-*anti*GII**.

One deficiency of all these complexes is related to their pronounced thermal sensitivity and instability in solution. To overcome this disadvantage, Collins and coworkers synthesized a series of new complexes by varying the nature of the *N*-alkyl substituents [23].The substitution of the *N*-Me group with larger *N*-benzyl or *N*-propyl substituent led to improved thermal and solution state stability. The new complexes, isolated as mixtures of *syn/anti* rotational isomers, were evaluated in desymmetrization reactions of *meso*-trienes and showed lower reactivities than the parent catalyst **19-*anti*GII**. Moreover, lower enantiomeric excesses in almost all cyclization reactions were registered, suggesting an important effect of the *N*-alkyl group on the observed enantioselectivities. On the other hand, the increased robustness of these catalysts allowed for their application also in more challenging metathesis reaction, such as the ARCM of prochiral trienes affording tetrasubstituted olefins. In the desymmetrization of trienes **30** and **32** catalyst **22-*anti*GII** (in which the *anti* isomer is the major isomer) gave **31** and **32** in 71% and 78% ee, respectively (Scheme 8) [28].

**Scheme 9:**
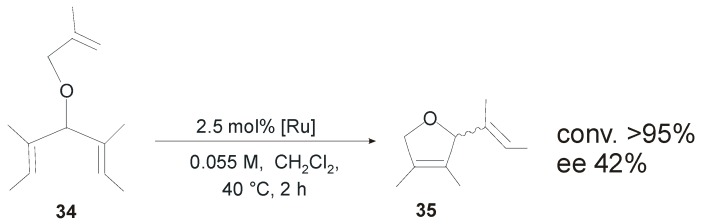
Asymmetric ring closing metathesis of **34** catalyzed by **11**-***anti*GII** and **11-*anti*HGII**.

Enantiopure catalysts **11-*anti*GII** and **11-*anti*HGII** (Figure 10), incorporating a *C_1_*-symmetric NHC ligand with *anti* phenyl groups on the backbone, were tested in the ARCM of prochiral trienes **18** and **34** (Scheme 9) [49]. In the ARCM of **18** both the catalysts gave quantitative conversion to **19** along with low enantiomeric excesses (18%–19%). The use of NaI as an additive led to higher ee values (52%–53% ee), as observed by Grubbs with chiral catalysts bearing *C_2_*-symmetric NHCs, and in contrast with the results reported by Collins with catalysts **19-*anti*GII**–**22-*anti*GII**, which are actually structurally much more similar to **11-*anti*GII** and **11-*anti*HGII**. In the challenging enantioselective desymmetrization of **34** to afford the tetrasubstituted cycloolefin **35** both the catalysts efficiently performed the cyclization of **34** (>95%), equaling the best results, in terms of conversion and enantioselectivity, obtained by Collins with modified versions of **19-*anti*GII**, in which the *N*-Me group is replaced with *N*-propyl group (95% conversion, 43% ee) [28].

**Figure 16:**
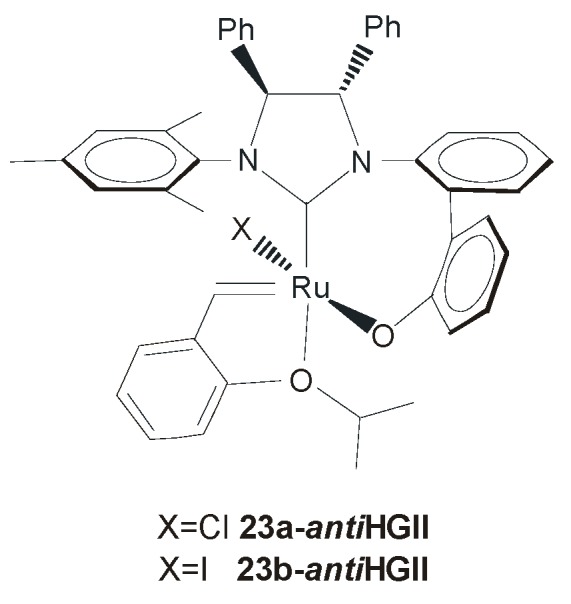
Catalyst **23-*anti*HGII** with chiral bidentate NHC.

The relevance of the chirality of the NHC backbone in ruthenium complexes with differing *N*-aryl substituents was also proved by the work of Hoveyda and co-workers. In 2005, they reported the synthesis of the biphenolate NHC complexes **23a-*anti*HGII** and **23b-*anti*HGII** (Figure 16), presenting a *C*_1_-symmetric bidentate NHC ligand [56].

Within these systems, the presence of *anti* groups on the NHC backbone influences the orientation of the achiral biphenyl moiety, which coordinates diastereoselectively to the ruthenium center. In this way, the chiral information on the NHC backbone is efficiently transferred to the metal. Although chromatographic isolation of chloride complex **23a-*anti*HGII** was not possible, both the catalysts **23a-** and **23b**-***anti*HGII** could be used *in situ* proving efficiency in a series of AROCM transformations with high enantioselectivities [56,57].

## 5. Chiral Ruthenium Complexes Bearing Backbone-Monosubstituted NHCs

**Figure 17:**
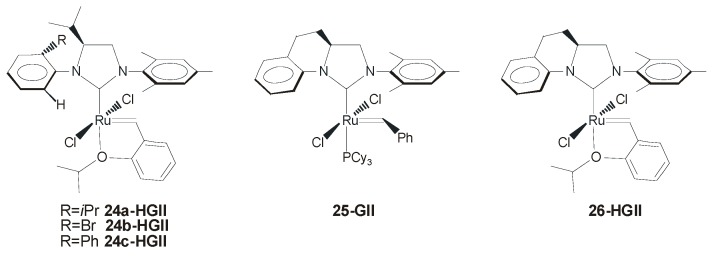
Catalysts with backbone-monosubstituted NHCs.

In 2010, a new concept in the design of chiral NHC Ru-based systems was introduced by Blechert and coworkers (Figure 17) [29]. The NHC ligand was characterized by a monosubstituted backbone with a single stereocenter, and two different *N*-aryl substituents. The mono-*ortho*-substituted phenyl group next to the stereocenter in the backbone efficiently transfers the chirality to the olefin coordination sphere, and the opposing mesityl group, due to the lack of backbone substituent, adopts a planar arrangement which increases the space for metathesis transformations. These catalysts proved to be highly stable and highly active, providing excellent results in terms of both enantioselectivity and *E*-selectivity in AROCM reactions. In the AROCM of **22** with 5 equivalents of styrene (Scheme 5), complex **24c-HGII** allowed for complete conversion of **22** also at low catalysts loading (0.05 mol %) or at low temperature (−10 °C), along with high enantiomeric excesses (88% and 93%, respectively) and *E/Z* ratios >30:1. Notably, no halide additives were required for this transformation.

**Scheme 10:**
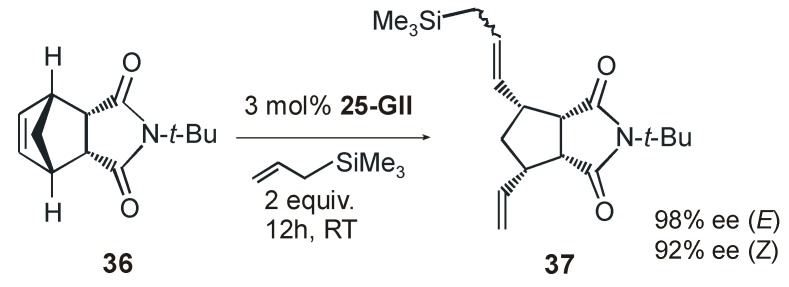
AROCM of **36** with allyltrimethylsilane in the presence of **25-GII**.

One year later the same group reported ruthenium catalysts **25-GII** and **26-HGII** coordinated with a new type of chiral NHC ligand presenting an intramolecular linkage between the *N*-aryl and the backbone which creates a rigid chiral environment around the metal [58]. Catalysts **25-GII** and **26-HGII** were employed in AROCM with excellent results in terms of activity, enantioselectivity, and substrate scope, however *E/Z* selectivities were less pronounced than those observed with previous complexes **24-HGII**. Significantly, for the first time the AROCM of norbornenes with allyltrimethylsylane as the cross-partner was investigated (e.g., AROCM of **36**, Scheme 10). The fixed, non–rotable *N*-aryl unit in the NHC ligand of catalysts **25-GII** and **26-HGII** led to higher enantioselectivities for both *E/Z* stereoisomers of the formed olefin with respect to Grubbs catalyst **18a-*anti*GII**, in which the possible partial *N*-aryl rotation gives rise to a more flexible reaction pocket, resulting in a lower enantioselectivity [59].

## 6. Conclusions

Since their discovery, the NHC-containing olefin metathesis ruthenium catalysts have undergone extensive modifications in the ligand shell with the purpose of providing effective catalysts that are readily available, easy to handle, reliable, and highly selective. Significant progress in catalyst design is undoubtedly related to the manipulation of the NHC framework. In this review we focused on the impact of NHC ligands bearing substituents at the backbone in a fixed stereochemical arrangement on the catalytic properties of metathesis ruthenium complexes. The reported results highlight the crucial role of NHC backbone configuration in giving positive enhancement for RCM reactions, especially with sterically demanding olefins, as well as for challenging asymmetric transformations. As a future perspective, we believe that the intriguing behavior of ruthenium complexes bearing unsymmetrical NHCs with different backbone configuration represent an important starting point for further development of metathesis catalysts.
